# Focal non-invasive deep-brain stimulation with temporal interference for the suppression of epileptic biomarkers

**DOI:** 10.3389/fnins.2022.945221

**Published:** 2022-08-17

**Authors:** Emma Acerbo, Aude Jegou, Charlotte Luff, Patrycja Dzialecka, Boris Botzanowski, Florian Missey, Ibrahima Ngom, Stanislas Lagarde, Fabrice Bartolomei, Antonino Cassara, Esra Neufeld, Viktor Jirsa, Romain Carron, Nir Grossman, Adam Williamson

**Affiliations:** ^1^Aix Marseille University:, INSERM, Institut de Neurosciences des Systèmes, Marseille, France; ^2^Department of Brain Sciences, Imperial College London, London, United Kingdom; ^3^Department of Epileptology, APHM, Timone Hospital, Marseille, France; ^4^Foundation for Research on Information Technologies in Society, Zurich, Switzerland; ^5^Department of Functional and Stereotactic Neurosurgery, Timone University Hospital, Marseille, France; ^6^Department of Medicine, Center for Bioelectronic Medicine, Karolinska Institute, Stockholm, Sweden

**Keywords:** temporal interference, deep brain stimulation, non-invasive stimulation, epilepsy, mouse model

## Abstract

**Introduction:**

Neurostimulation applied from deep brain stimulation (DBS) electrodes is an effective therapeutic intervention in patients suffering from intractable drug-resistant epilepsy when resective surgery is contraindicated or failed. Inhibitory DBS to suppress seizures and associated epileptogenic biomarkers could be performed with high-frequency stimulation (HFS), typically between 100 and 165 Hz, to various deep-seated targets, such as the Mesio-temporal lobe (MTL), which leads to changes in brain rhythms, specifically in the hippocampus. The most prominent alterations concern high-frequency oscillations (HFOs), namely an increase in ripples, a reduction in pathological Fast Ripples (FRs), and a decrease in pathological interictal epileptiform discharges (IEDs).

**Materials and methods:**

In the current study, we use Temporal Interference (TI) stimulation to provide a non-invasive DBS (130 Hz) of the MTL, specifically the hippocampus, in both mouse models of epilepsy, and scale the method using human cadavers to demonstrate the potential efficacy in human patients. Simulations for both mice and human heads were performed to calculate the best coordinates to reach the hippocampus.

**Results:**

This non-invasive DBS increases physiological ripples, and decreases the number of FRs and IEDs in a mouse model of epilepsy. Similarly, we show the inability of 130 Hz transcranial current stimulation (TCS) to achieve similar results. We therefore further demonstrate the translatability to human subjects *via* measurements of the TI stimulation vs. TCS in human cadavers. Results show a better penetration of TI fields into the human hippocampus as compared with TCS.

**Significance:**

These results constitute the first proof of the feasibility and efficiency of TI to stimulate at depth an area without impacting the surrounding tissue. The data tend to show the sufficiently focal character of the induced effects and suggest promising therapeutic applications in epilepsy.

## Highlights

- A non-invasive deep brain stimulation applied *via* temporal interference achieves to target the hippocampus in mice and human cadavers.
- TI stimulation at the beating frequency of 130 Hz is able to suppress epileptic biomarkers of epilepsy in a mouse model of epilepsy.
- TI stimulation can target a deep structure in the human brain (hippocampus) with a limited field in the surrounding cortex and structures.

## Introduction

Epilepsy is a severe and highly prevalent neurological disease, affecting about 1% of the population worldwide (Fiest et al., 2017). Despite the continuous development of new antiseizure medications (Löscher and Schmidt, 2011), pharmacoresistance remains a major issue for about one-third of patients, who may benefit from epilepsy surgery when epilepsy is focal (Ryvlin et al., 2014). For patients with drug-resistant epilepsy not eligible for surgery or with failure of resective surgery, neuromodulation, that is, electrical stimulation, be it invasive or not, provides an alternative option. Among neuromodulation techniques, deep brain electrical stimulation (DBS) has been increasingly explored and included in clinical practice (Velasco A. L. et al., 2007; McLachlan et al., 2010). The most common methods of DBS use implanted leads targeting the thalamus, hippocampus, or other parts of basal ganglia depending on the indication and types of epilepsies (Zangiabadi et al., 2019). These approaches proved to be efficient, effective, and well-tolerated (Li and Cook, 2018), but remain invasive and are not deprived of possible complications (e.g., neurological deficit, hematoma, and infection) (Pilitsis et al., 2008). As a result, non-invasive neurostimulation techniques, such as transcranial current stimulation (TCS), provide interesting perspectives and are currently on trial as therapeutic approaches in epilepsy (Sudbrack-Oliveira et al., 2021). However, the limited spatial accuracy and the low penetration of deepest brain regions are limitations of current TCS protocols (Brunoni et al., 2012). To address this, we utilize the method of temporal interference (TI) stimulation, a focal stimulation method that has the ability to stimulate deep brain structures with interferences at points located at a significant distance from the surface electrode (Grossman et al., 2017). This can be achieved by the use of two high frequencies (>1 KHz) which are known to not have an impact on the neurons (Hutcheon and Yarom, 2000). The two frequencies have to have an offset which will be the frequency of the DBS stimulation.

Ideal parameters for neuromodulation regimes specifically treating epilepsy using electrical stimulation of the peripheral (Ben-Menachem, 2002) or central nervous system (CNS) (Davis and Gaitanis, 2020) have been evaluated in both animal and clinical studies (Schulze-Bonhage, 2017). The aim of CNS stimulation in epilepsy is to target a key region in the brain and apply electrical stimulation, traditionally with a DBS electrode, to suppress seizures and the associated epileptogenic biomarkers (Li and Cook, 2018). Inhibitory DBS is delivered at high-frequency stimulation (HFS), typically between 100 and 165 Hz (Theodore and Fisher, 2004). The main DBS study for seizure suppression in epilepsy was the SANTE trial, with more than 100 patients enrolled. It aimed to assess the safety efficacy of stimulation of the anterior nucleus of the thalamus (ANT). The trial has shown that HFS at 145 Hz could significantly decrease seizure frequency and improve patients’ quality of life (Fisher et al., 2010; Salanova et al., 2015). Originally HFS at 130 Hz was a parameter used in the treatment of essential tremor and Parkinson’s disease; however, it has also been successfully applied in epilepsy (Benabid et al., 2001). The same frequency has now been used effectively in several regions of the brain, such as the anterior nucleus of the thalamus (Krishna et al., 2016), the centromedian nucleus of the thalamus (Velasco F. et al., 2007), or thehippocampus (Vonck et al., 2013). More specifically in the hippocampus, it has been shown that 130 Hz decreases the number of seizures in patients with mesio-temporal lobe epilepsy (MTLE) (Velasco et al., 2001; Cukiert et al., 2017). Furthermore, numerous studies in rodents have also shown the effectiveness of 130 Hz stimulation on seizures and thresholds to evoked seizures in the hippocampus (Wyckhuys et al., 2007, 2010).

In the work presented here, we wanted to analyze if the provided TI stimulation would be able to decrease epileptic biomarkers in the hippocampus of a mouse model of epilepsy. Finally, we also wanted to investigate if the TI Stimulation applied to a human head would also target a deep structure. Thus, we stimulated human cadavers with electrodes placed on the skin and recorded the resulting fields inside the head with stereo-electro-encephalography (SEEG) electrodes.

## Materials and methods

### Electromagnetic exposure modeling

Electric exposure simulations have been performed using the structured ohmic-current-dominated electro-quasistatic finite-element-method solver from Sim4Life (ZMT Zurich MedTech AG, Switzerland), which has been verified to suitably approximate Maxwell’s equations for the frequencies and setup of interest. The highly detailed MIDA anatomical head model (Iacono et al., 2015) that distinguishes > 130 different anatomical regions was used, and the two applied currents were simulated individually by applying corresponding Dirichlet voltage boundary conditions to the respective electrode pairs and normalizing to the resulting total current. Electrical conductivity values were assigned to the different tissues (Hasgall et al., 2012; McCann et al., 2019). To simulate the impact of stereotaxic electroencephalographic electrodes (SEEG) presence in terms of local field enhancement due to the presence of highly conductive metal contacts, CT images from a patient were co-registered with the MIDA model using Sim4Life and ensured a good match between skulls. Thresholding of the metal-related image artifacts in CT scan was used to guide the placement of 14 SEEG lead models featuring 13–17 cylindrical electrode contacts each (modeled as perfect electric conductors) separated by insulating segments according to manufacturer specifications. Rectilinear discretization with a resolution of 0.27–0.65 mm (maximal refinement at the SEEG contacts) was employed (142 million voxels).

The following quantities were extracted for analysis and visualization using native Sim4Life postprocessing functionalities, as well as custom Python scripts: maximal TI modulation amplitude and peak high-frequency (HF) field magnitude according to the equations from Grossman et al. (2017) their normal component on the brain surface (as a measure for cortical TI and HF stimulation), overall and hippocampal peak, peak 2 mm-averaged [according to ICNIRP Guidelines on the exposure safety standard (International Commission on Non-Ionizing Radiation Protection [ICNIRP], 2013)], and isopercentile values.

### Animals

All experiments were performed in accordance with European Council Directive EU2010/63 and French Ethics approval (Williamson, n. APAFIS#20359-2019041816357133 v10). Animals were kept in transparent cages in groups of three to five, in a temperature-controlled room (20 ± 3°C) with a 12/12-h night-day cycle. All animals had *ad libitum* access to food and water.

### Surgical procedure

For this study, we used 29 male OF1 mice (Charles Rivers Laboratories, France) aged 8–10 weeks. Mice were implanted with two pairs of minimally invasive cortical electrodes and with an intracerebral depth-electrode in the hippocampus. A depth-electrode was used to perform a standard kindling protocol and remained in place to record the electrical activity. Mice were divided into groups; all underwent a surgical procedure and kindling protocol. About 12 mice received TI-HFS (f1:1,300 Hz, f2:1,430 Hz), 9 were sham, and 8 mice received CT-HFS (130 Hz).

Mice were anesthetized *via* an intraperitoneal injection of ketamine (50 mg/kg) and xylazine (20 mg/kg) and placed in a stereotaxic frame. After midline scalp incisions, the following stereotaxic coordinates were used for craniotomies: Cortical electrodes [AP: −1.94, ML: +0.5; −0.5; −3.9; −4.3] and Implantable twisted-pair platinum electrodes (from PlasticsOne; wire length = 5 mm, individual wire diameter = 125 μm) [AP: −2.7, ML: +2.04, DV: 1.30] (Paxinos Atlas) using a 20° angle to reach the hippocampus by considering the constraints due to the location of the minimally invasive cranial electrodes. The coordinates for the cortical electrodes were calculated with the Finite Element Model (Comsol) in a study already published (Missey et al., 2021). Then, four screws (Component Supply, Miniature Stainless Steel Screws: TX00-2FH) were placed on the cortex without penetration into brain tissue. Subsequently, dental cement (Phymep, SuperBond) was applied to the skull surface to fix the screws. After surgery, all mice were kept in separate cages to avoid fighting and damage to implanted electrodes and were observed for signs of pain, distress, and neurological complications.

### Electrical stimulation and recordings

All recordings were done with a recording and stimulation controller (RHS Stim/Recording controller, Intan^®^, Los Angeles, CA, United States) and stimulation was done by either the RHS recording and stimulation controller (kindling protocol) or a function generator (Keysigth^®^, Santa Rosa, United States) driving a DS5 current source (Digitimer©, London, United Kingdom) to reach the desired current. To process the data, all files have been converted from RHS format to a readable format for us using MATLAB format and the specific format of AnyWave, a visualization software for electrophysiological data (Colombet et al., 2015).

#### *In vivo* experiments

After 7 days of recovery following surgery, the protocol for Rapid Kindling was done on all mice after a baseline recording (Supplementary Figure 1). This procedure evoked in mice epileptogenic biomarkers, such as pathological IEDs, and has been previously described in this study (Musto et al., 2009). Shortly, a 50 Hz stimulation has been done by the electrode placed in the hippocampus (bipolar and biphasic) at 50 μA. Then, the current has been ramped up until eliciting an afterdischarge (AD). Then, this stimulation was repeated over 2 days to get an epileptic state for 24 h. Directly after the last session of kindling, TI stimulation was provided by the DS5s and driven at 1,300 Hz and 1,430 Hz by the function generator. The two frequencies applied for TI were square pulses, like those used in the clinic, called here as Pulse Width Modulated (PWM)-TI. This stimulation was biphasic pulses of 100 μs with pulse amplitudes of 60%*AD threshold μA for 1 h. For CT-HFS, 130 Hz has been directly applied by one pair of cortical electrodes with the same amplitude and duration as TI-HFS treatment.

### Human cadavers

Two anatomical subjects were provided by “*service des corps donnés à la science*” by Aix Marseille University and all experiments were performed in the Faculty of Medicine *La Timone* (Aix Marseille University). All subjects were perfused with zinc chloride and stored in the freezer until experiments. Subjects were left at least 2 h at 20°C before any stimulation/recording session. For depth recording, 12 SEEG electrodes (Alcis©, France) were implanted mainly in the temporal lobe on both sides and for scalp stimulation, and classical ECG electrodes were placed on the skin (Ambu^®^). All SEEG insertions were performed by a neurosurgeon (RC) based on his surgical expertise in SEEG for adult patients. The implantation was based on mere anatomical landmarks on the entry point without stereotactic reference. For electrodes localization, subjects’ heads were cut and a CT scan of the head was performed after the experiments at CERIMED, Aix Marseille University, France.

### Analysis

#### *In vivo* experiments

To detect events, we performed a semi-automatic detection on the signal. Automatic detection was processed by using Delphos software, a detector of IEDs and oscillations used mainly in clinical research (Roehri et al., 2018). Delphos is based on a method of whitening (*Z*_*H_0*_) the time-frequency spectrum to optimize signal to noise ratio at each frequency. Events of interest are detected while they are above a specific threshold in the spectrum. Before applying Delphos on the signal, it was down-sampled at 3,750 Hz. Once events were detected, they were reviewed by an expert (EA). Events were accepted/rejected by the expert. Finally, events (IEDs [15–45]Hz, SPW-Rs[150–250]Hz, and FRs[250–500]Hz) were extracted in MATLAB format to perform an average of all events and determine the duration, amplitude, and rates by minutes. For pathological FRs, suppression of epileptic markers has been calculated using a ratio (PostTreatment*100/PreTreatment) to show the effect of our treatment groups.

#### Human cadavers

To ensure correct anatomical position of SEEG electrodes, a CT scan of the cadaver’s head was performed. Then, we used Gardel, to localize and label SEEG contacts (Medina Villalon et al., 2018). To analyze the intra-cerebral signal, we used MATLAB, especially the Signal Processing toolbox, to determine the envelope of stimulation induced by TI. First, the signal was filtered with a band pass filter with a passband frequency of [1000–3000] for TI. Second, the magnitude of its analytics signal (envelope) was calculated on the filtered signal by using Hilbert transformation. A sliding window was used to determine the amplitude of the envelope peak-to-peak in mV, by subtracting the minimum of the envelope from the maximum. The median value of all the windows was used as the amplitude value for the contact. This process has been performed for each contact which allows having an amplitude value by contact. Finally, the amplitude value was projected on the electrodes in the mesh of the anatomical image (MNI template) to visually determine which part of the brain was stimulated.

### Statistical analysis

This study has been designed so that all statistical tests have a power of 80%. After gathering recordings for all mice, FRs, IEDs, and SPW-Rs were analyzed using R studio^®^. To adapt the test to the data distribution, all groups were tested for normal distribution using Shapiro tests. As distributions were not normal, Wilcoxon rank tests were performed to highlight differences between the different groups (Alpha = 5%).

## Results

### Temporal interference stimulation can focally target the hippocampus in both mice and humans

**FIGURE 1:**
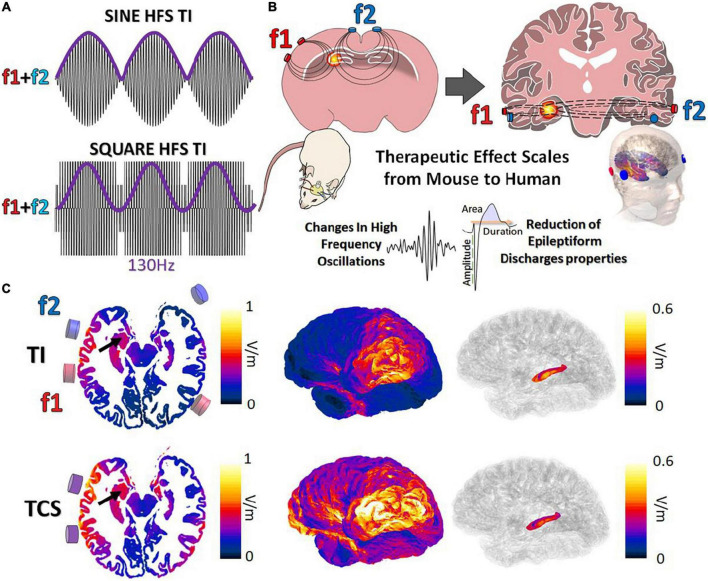
Forms of temporal interference and ability to scale to larger subjects. **(A)** Classically, TI has been created *via* a combination of 2 kHz sine waves (f1:1,300 Hz f2:1,430 Hz, envelope = 130 Hz). We investigated the impact of TI by mixing square waves (PWM-TI). **(B)** To provide TI stimulation, cortical electrodes (2 pairs) were placed on the cortex of mice and human skin. In both cases, the aim was to focally reach one side of the hippocampus and provide stimulation at depth. **(C)** Simulated TI envelope modulation amplitude distributions (along the direction of maximal modulation) and peak carrier field magnitude (bottom; TCS) and their corresponding surface field views. Arrows: hippocampus.

Classically, TI is obtained *via* the combination of 2 kHz sine waves (Grossman et al., 2017; Figure 1A). Although effective, the majority of clinical stimulation paradigms utilize square pulses for stimulation applications. In the work presented here, we aimed to demonstrate the possibility to target, in human or mice brains, and to stimulate the hippocampus with a tunable frequency. The two stimulation pairs have been oriented differently to reflect the specific orientations of the mouse hippocampus (coronal) and human hippocampus (axial) (Figure 1B). The two frequencies used here are f1:1,300 Hz and f2:1,430 with Δf = 130 Hz to perform a DBS in a non-invasive way. To determine where to place the cortical electrodes to evoke stimulation in the human hippocampus, electromagnetic (EM) modeling using the finite element method (FEM) has been performed. Simulations were performed with (Supplementary Figures 2A,B) and without (Figure 1C) inclusion of SEEG electrodes (image-based placement) in an anatomical head model, for TI stimulation and TCS. When comparing TI-HFS and TCS exposure distributions, it appears that TI-HFS stimulation does not impact the hippocampus overlaying cortex and brain structures as much as TCS does. Thus, we wanted to see if we could have an impact on physiological hippocampal activities, starting with the murine hippocampus.

### Temporal interference stimulation can influence hippocampal brain rhythms in mice

**FIGURE 2:**
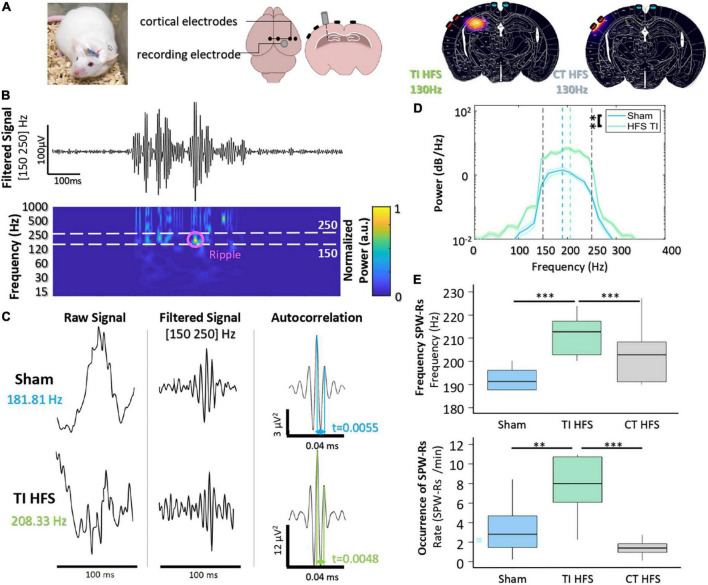
Focal hippocampal stimulation induces SPW-Rs. **(A)** Mice are implanted with depth-electrode to induce epileptiform activity. Finite element model of TI-HFS and CT-HFS. It shows the creation of a hot spot of stimulation in the hippocampus *via* TI compared to the cortical stimulation with HFS. **(B)** Raw recording and time-frequency plot of activity in the hippocampus showing the identification of SPW-Rs in the frequency band [150–250 Hz]. **(C)** Example SPW-Rs and the averaged autocorrelation from Sham and TI-HFS. The distance in time of the first and second peaks of the average autocorrelation shows SPW-Rs’ frequency. **(D)** Analysis of the power spectral density to get the frequency of the recorded SPW-Rs (^**^p-value < 0.01). **(E)** Frequency and occurrence of SPW-Rs for TI-HFS (green), Sham (blue), and CT-HFS (gray) (^***^p-value < 0.001).

To analyze the non-invasive DBS-like effect of stimulation (TI-HFS), we compared its impact with a cortical high-frequency stimulation at 130 Hz (CT-HFS) and a non-treatment group (Sham) on hippocampal electrophysiological biomarkers. The distribution of the envelope modulation amplitude has been calculated using FEM and compared with the field distribution of CT-HFS. According to the simulation, CT-HFS cannot activate the hippocampus without activating the cortex earlier, whereas with TI-HFS, it is possible to stimulate the hippocampus and spare the cortex (Figure 2A).

Hippocampal sharp wave–ripple complexes (SPW-Rs) are observed in both slow-wave sleep and awake behavior during inactivity and are considered to be involved in the transfer of stored information to the neocortex for memory consolidation (Buzsáki, 2015). SPW-Rs are oscillations that are visible in a time frequency plot in [150–250] Hz band (Figure 2B). Behrens et al. (2005) first demonstrated that it is possible to evoke SPW-Rs *via* an implanted device and that their properties change depending on whether SPW-Rs are physiological or induced.

First, we observed that there are differences between the properties of SPW-Rs (sham) and focally induced (TI-HFS) SPW-Rs (Figure 2C). The averaged autocorrelation of SPW-Rs from the two cohorts allowed us to determine the frequency of the detected SPW-Rs. We demonstrated a clear shift in SPW-Rs’ frequency induced with focal TI-HFS (from, respectively, 181 to 208 Hz). In addition, the power spectral density of the average confirmed this shift in SPW-Rs’ frequency. However, it was also observed a significant increase in SPW-Rs strength for induced SPW-Rs (Sham vs. TI HFS *p*-value = 0.003) (Figure 2D). CT-HFS had not the same impact on SPW-Rs, where SPW-Rs’ frequencies and occurrence for Sham and CT-HFS were equivalent. This may indicate that SPW-Rs analyzed were physiologic and not induced by the stimulation (Sham vs. TI HFS *p*-value < 0.001; Sham vs. CT HFS *p*-value = 0.073; CT HFS vs. TI HFS *p*-value < 0.001). Additionally, only TI-HFS increased SPW-Rs’ incidence (SPW-Rs/min), where TI-HFS can multiply by 4, the number of SPW-Rs detected (Sham vs. TI HFS *p*-value = 0.003; CT HFS vs. Sham *p*-value = 0.278; TI HFS vs. CT HFS *p*-value < 0.001) (Figure 2E).

Taken together, these results indicated the ability of TI-HFS to achieve stimulation of the hippocampus, equivalent to previous studies evoking similar results with direct “*in situ*” depth stimulation. Such effect was not observed with a transcranial CT-HFS. We then analyzed the anti-epileptic effect of TI-HFS by recording the occurrence of interictal epileptiform discharges (IEDs) and Fast Ripples (FRs) [250–500 Hz] in a mouse model of epilepsy.

### High-frequency stimulation temporal interference at 130 Hz decreases the expression of epileptogenic biomarkers

A key feature to assess the ability of therapeutic stimulation of seizures is to analyze its impact on interictal epileptogenic biomarkers. One of the most well-known interictal biomarkers of epileptogenicity is IEDs. MTLE patients treated with HFS at 130 Hz from DBS electrodes in the hippocampus had seizure frequency reduced and this was correlated with a reduction of IEDs (Boon et al., 2007). A second biomarker consists of high-frequency oscillations, FRs, approximately 250–500 Hz (Ibarz et al., 2010). FRs are often associated with IEDs (Jiruska et al., 2010; Wang et al., 2013), and their surgical resection is correlated with post-surgical seizure freedom (Akiyama et al., 2011). Similarly, DBS of the hippocampus in epilepsy can also decrease the occurrence of FRs in patients and rodents (Mălîia et al., 2017; Lévesque et al., 2019).

**FIGURE 3:**
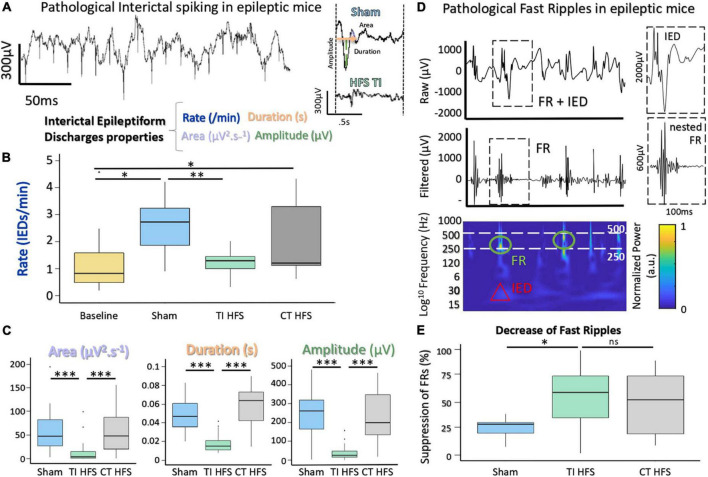
Suppression of IEDs and FRs with a non-invasive DBS *via* TI-HFS. **(A)** Pathological IEDs and their properties: rate, duration amplitude, and area of the wave. **(B)** Occurrence of pathological IEDs in mice hippocampus. Baseline (yellow) grouped all mice before kindling induction (*p-value < 0.05). **(C)** Analysis of IEDs properties. area, duration, and amplitude were calculated for detected IEDs. **(D)** Raw and filtered [250–500] Hz signal with detected FRs. Time-frequency plot showing activation in FRs band. **(E)** Pathological FRs occurrence. A ratio for each mouse was calculated to compare pre- and after-treatment. ***p-value < 0.001 and **p -value < 0.01.

To assess the impact on TI-HFS, baseline recordings were made in all mice before undergoing the kindling protocol (Supplementary Figure 1). Traditional analysis of IEDs evaluates several components, such as rate, area under the wave, duration of IEDs, and amplitude (Figure 3A; Huneau et al., 2013). For rate, IEDs recorded after TI-HFS occurred less frequently (decreased by a factor of 3 compared with Sham). Only TI-HFS was able to reduce the number of IEDs to a level similar to before the induction of epilepsy—Baseline (Sham vs. TI HFS *p*-value = 0.001; Baseline vs. CT HFS *p*-value = 0.041; Baseline vs. Sham *p*-value = 0.005; CT HFS vs. TI HFS *p*-value = 0.384; CT HFS vs. Sham *p*-value = 0.401; Baseline vs. TI HFS *p*-value = 0.287). Interestingly, it appears that there is no statistical difference between TI-HFS and CT HFS on the IEDs rate (Figure 3B).

For the analysis of IEDs’ characteristics, TI-HFS significantly decreased the area under the wave, total duration, and amplitude of IEDs.

In all cases, TI-HFS gave rise to a significant reduction of epileptic biomarkers, while CT-HFS and Sham were unable to achieve such effects (Area Sham vs. TI HFS *p*-value < 0.001; CT HFS vs. TI HFS *p*-value < 0.001; CT HFS vs. Sham *p*-value = 0.761 Duration Sham vs. TI HFS *p*-value < 0.001; CT HFS vs. TI HFS *p*-value < 0.001; CT HFS vs. Sham *p*-value = 0.183; Amplitude Sham vs. TI HFS *p*-value < 0.001; CT HFS vs. TI HFS *p*-value < 0.001; CT HFS HFS vs. Sham *p*-value < 0.001) (Figure 3C).

The second epileptogenic biomarker analyzed was FRs, an indicator of epileptogenicity used in the identification of the epileptogenic zone. FRs are oscillations that can be detected after filtering the raw signal, typically between [250 and 500 Hz]. Interestingly, FRs very often co-occur with IEDs which has been stated as a good indicator for epileptogenicity (Roehri et al., 2018; Figure 3D). We analyzed the change in FRs, a ratio before and after treatment (or no treatment in case of sham) was performed. For TI-HFS, a clear decrease in the number of FRs was seen with more than 50% FR’s suppression. For CT-HFS stimulation, there was no significant impact on the reduction of FRs compared to Sham even if there were no significant differences compared to TI-HFS (Sham vs. TI HFS *p*-value = 0.023; CT HFS vs. TI HFS *p*-value = 0.327; CT HFS vs. Sham *p*-value = 0.132) (Figure 3E).

Overall, these results illustrated the impact of TI-HFS, upon two biomarkers of epilepsy within the hippocampus, reducing both features and number of IEDs and prevalence of FRs.

### Scaling high-frequency stimulation temporal interference: From mouse to human brain

**FIGURE 4:**
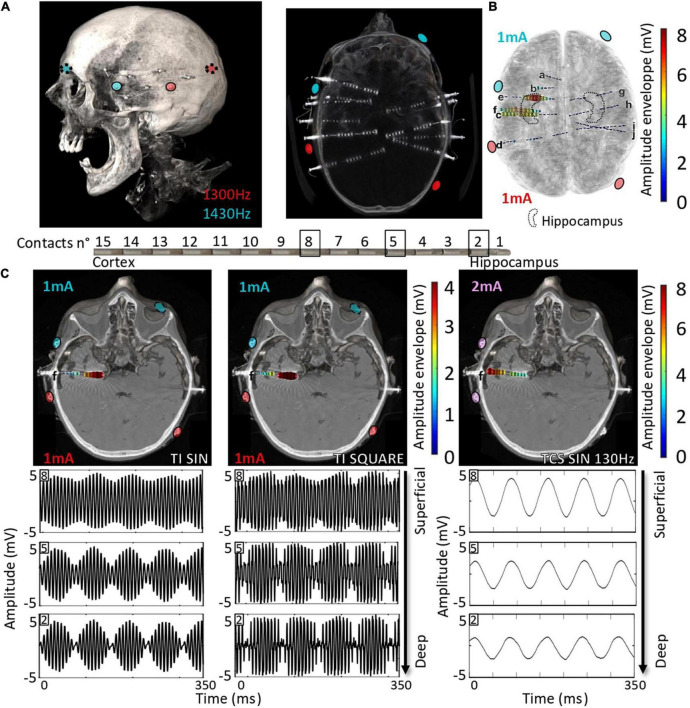
Scaling the effect of TI-HFS at 130 Hz in the human head. **(A)**. Placement of electrodes on the skin of the human cadaver. About 10 SEEGs were implanted in the brain for MTLE patients to record the stimulation potentials inside the brain. **(B)** Co-registration with the scanner and a template MRI show the amplitude of the envelope recorded during the TI-HFS session. It gives an indication on where the focus of stimulation is and the amplitude of the envelope of stimulation. Here, 1 and 3 mA were chosen to better target the anterior hippocampus in this specific cadaver and the field modulation envelope can reach an amplitude of 7 mV. **(C)** Electrode f is used to show the depth of the focal stimulation of TI-HFS in both square and sine waves. For TCS-like 130 Hz, it shows stronger stimulating fields in the cortex, which is primarily activated, compared to the deep structures, unlike for TI with sine waves or square waves (PWM TI).

Here, we explored the possibility of applying non-invasive TI stimulation targeting the hippocampus in humans by analyzing TI-HFS stimulation in human cadavers. Several SEEG electrodes were implanted in the temporal lobe of cadavers to record the applied stimulation potential (Figure 4A). By mixing 1,300 and 1,430 Hz with electrodes placed on the skin, a focus of stimulation in the anterior part of the hippocampus was created as previously done in mice. The amplitude of the envelope of stimulation was at 8 mV only in the hippocampus. The two other electrodes placed in the central area (electrodes c and f) recorded an envelope amplitude of around 5 mV. Electrodes placed outside of the hippocampus and contralateral SEEG electrodes did not record any amplitude modulated signal (Figure 4B). This result confirmed that TI can reach deep-seated structures, here the hippocampus, in human cadavers, and shows that scaling of TI-HFS as previously done in mice is achievable in a human subject. To further analyze the effect of the PWM signal used across the study (TI-HFS), we compared its effect with traditional TI with sine waves. We show an example electrode (f) to illustrate the main differences between the two forms of TI and additionally include transcranial stimulation (TCS) at 130 Hz using sine waves. The modulation envelope amplitudes increase at depth in the cases of square and sine TI as is shown on the raw recording in the bottom right panels (Figure 4C). Between contacts n°8 (superficial) and n°2 (deepest), the amplitude of the envelope (upper part of the recorded signal) is multiplied by 4. Looking at TCS, only the cortex gets stimulated, and the amplitude of the signal decreases with depth.

In our human cadaver experiments, we used SEEG electrodes to record the potential created by TI-HFS. We showed that by stimulating with TI, a significant envelope amplitude is recorded at depth, and it decreased on the electrodes’ contacts placed in the cortex. This is not the case when 130 Hz is applied *via* TCS where the largest amplitude is recorded at shallow.

## Discussion

Essentially, the results presented here are consistent with the above-mentioned studies demonstrating a positive effect of HFS DBS on the hippocampus. First, we performed Finite Element Method (FEM) simulations in both mice and humans. Then, by using TI from electrodes on mice’s cortex, we showed the impact on epileptic biomarkers (IEDs, FRs) with a focal 130 Hz stimulation created with PWM-TI. Along these lines, we showed the ability to scale this technique in human cadavers implanted with SEEG electrodes to record the TI fields. In all applications, our PWM-TI stimulation was compared with conventional HFS from the cortex which failed to reach deep structures and change the hippocampus epileptic biomarkers.

For patients with intractable epilepsy not eligible for surgery or with failure of previous resective surgery, neuromodulation techniques turn out to be promising options (Vonck et al., 2002; Krishna et al., 2016). These techniques encompass invasive (e.g., DBS) and non-invasive stimulations (e.g., TCS). A complementary non-invasive stimulation that could provide similar results would be undoubtedly very useful. Some TCS methods are already promising and show some degree of seizure reduction, particularly those using tDCS (San-Juan et al., 2017). However, TCS is probably limited by the depth of penetration and spread of the applied electric fields. The results of this study appear encouraging and prompt to develop and refine TI as a new TCS paradigm capable of modulating the activity of deep structures (hippocampus in this study).

Simulations revealed that local field enhancement related to the presence of metallic SEEG contacts affects both TCS and TI exposure—for TI exposure, an important factor is that field lines from both channels are concentrated near and perpendicularly oriented to a highly conductive material, thus maximizing TI interference. However, this effect is highly localized, affecting < 1‰ of the brain volume significantly (Supplementary Figure 2C). These simulations do not account for field pickup by SEEG lead that could result in further field increase and power deposition near electrodes—proper lead characterization, for example, using methodologies from ISO (2018) and Liorni et al. (2018), would be required to examine the stimulation and safety relevance of this effect.

Mouse brain stimulation experiments in this work have demonstrated that superficial cortical TI-HFS, but not CT-HFS, is able to modulate physiological and pathophysiological activities of the hippocampus. For healthy physiological activity, it is understood that focal electrical stimuli can induce long-term potentiation (LTP), the neurophysiological process underpinning learning and memory, and will lead to the generation of SPW-Rs in the hippocampus (Jiang et al., 2018). In mice, TI-HFS can evoke SPW-Rs, which have higher oscillation frequencies compared to intrinsic SPW-Rs recorded in Sham conditions, replicating the results of focal stimulation of the same hippocampal region with depth-electrodes. In contrast, CT-HFS applied to the cortex was not able to modify the hippocampus activity. Here, we showed an ability of focal TI to influence natural brain rhythms by shifting central frequencies, strength, and incidence of SPW-Rs, never seen with non-invasive direct hippocampal stimulation.

For pathophysiological activity in mice, TI-HFS was shown to decrease both FRs and IEDs. In the case of IEDs, the spike rate returned to baseline (before epilepsy induction) and can also significantly reduce epileptic features of IEDs. Traditional transcranial methods of stimulation are not completely ineffective, and in the study here, we see that HFS applied to the cortex has an effect sized between TI-HFS and Sham. CT-HFS is not as focal and its effect is not as dramatic, which is in line with the literature (Brunoni et al., 2012).

The results in mice have then been scaled to humans. In our human cadaver experiments, we used SEEG electrodes to record the potential created by TI-HFS. We demonstrated that we obtained larger electrical amplitudes in depth when using TI and reduced stimulation amplitudes in the surrounding cortex. This is not the case when 130 Hz is applied *via* conventional TCS stimulation. We showed that the cortex near the electrodes has the largest amplitude of stimulation and that the recorded electric field unsurprisingly decreases with depth. However, the main difference between our rodent and human experiments is the invasiveness of the stimulating electrodes. In mouse experiments, the electrodes’ position was minimally invasive to avoid any motion during freely-moving-awake behavior. For humans, skin electrodes were used on the scalp, creating a fully non-invasive deep brain stimulation.

## Conclusion

In summary, TI, as a completely non-invasive stimulation method, was able to achieve deep brain structure (hippocampus) in both mice and human cadavers. As such, it offers a new perspective on non-invasive neuromodulation techniques for patients with refractory epilepsy ineligible for surgery or with failure of the latter and need of stimulation of deepest brain structures. TI could significantly change the manner in which neuromodulation in epilepsy is delivered because it can be no longer necessary to receive an invasive implant. A minimally invasive form of TI therapy could be prescribed (e.g., subcutaneous electrodes) or an intermittent, totally non-invasive therapy could be tested for efficacy before a potential DBS device implantation.

## Data availability statement

The raw data supporting the conclusions of this article will be made available by the authors, without undue reservation.

## Ethics statement

This study involving animals was reviewed and approved by the Ethics committee of the University of Aix-Marseille and signed by the French Ministry of Higher Education, Research and Innovation. Williamson, n. APAFIS#20359-2019041816357133 v10.

## Author contributions

AW conceived the project with input from NG and EA. EA, SL, FM, and BB performed experiments. BB, EN, and AC performed finite-element simulations. CL and PD provided input to the experiments. EA and AJ analyzed neural data. AW and EA wrote the manuscript with input from the other authors, including RC, FB, and VJ. All authors contributed to the article and approved the submitted version.

## Supplementary material

Supplementary Figure 1 Application of TI on human and mice. **(A)** Epilepsy in mice has been induced *via* a kindling model. Then, PMW TI has been applied for 1 h and analysis has been performed on the epoch before and after the TI-HFS. **(B)** In cadavers, TI (sin and square at 130 Hz) has been applied for 2 min each. **(C)** Characteristics of the rapid kindling model of induction of epilepsy used.

Supplementary Figure 2 EM simulations reveal highly localized field enhancement by the presence of metallic SEEG electrodes. **(A)** Illustration of the head models with the SEEG leads and the emplacement of the stimulation electrodes. **(B)** Simulated temporal interference envelope modulation amplitude distributions (left: TI along the direction of maximal modulation) and peak carrier field magnitude (right) in the presence of the SEEG leads, at a normalized current of 1 mA per channel. **(C)** Cumulative histograms of the cortical and hippocampal TI modulation and peak carrier distributions.
