# Supplementary figures and images for: Focal non-invasive deep-brain stimulation with temporal interference for the suppression of epileptic biomarkers
Source: Front Neurosci. 2022 Aug 17;16:945221. doi: 10.3389/fnins.2022.945221 (PMC9431367; doi:10.3389/fnins.2022.945221)

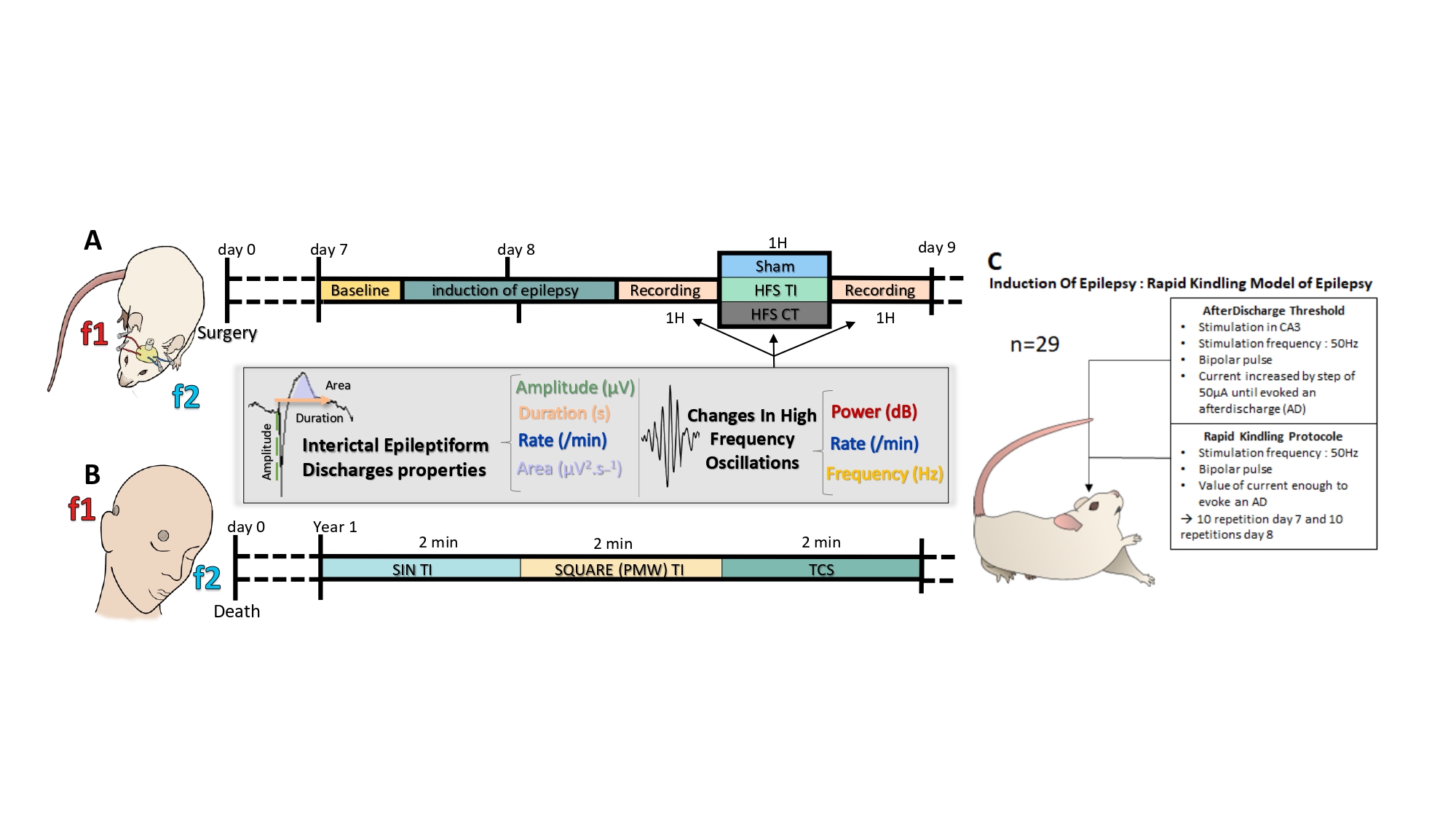

Supplement: Supplementary Figure 1 — Application of TI on human and mice. (A) Epilepsy in mice has been induced via a kindling model. Then, PMW TI has been applied for 1 h and analysis has been performed on the epoch before and after the TI-HFS. (B) In cadavers, TI (sin and square at 130 Hz) has been applied for 2 min each. (C) Characteristics of the rapid kindling model of induction of epilepsy used. [file Image_1.JPEG]

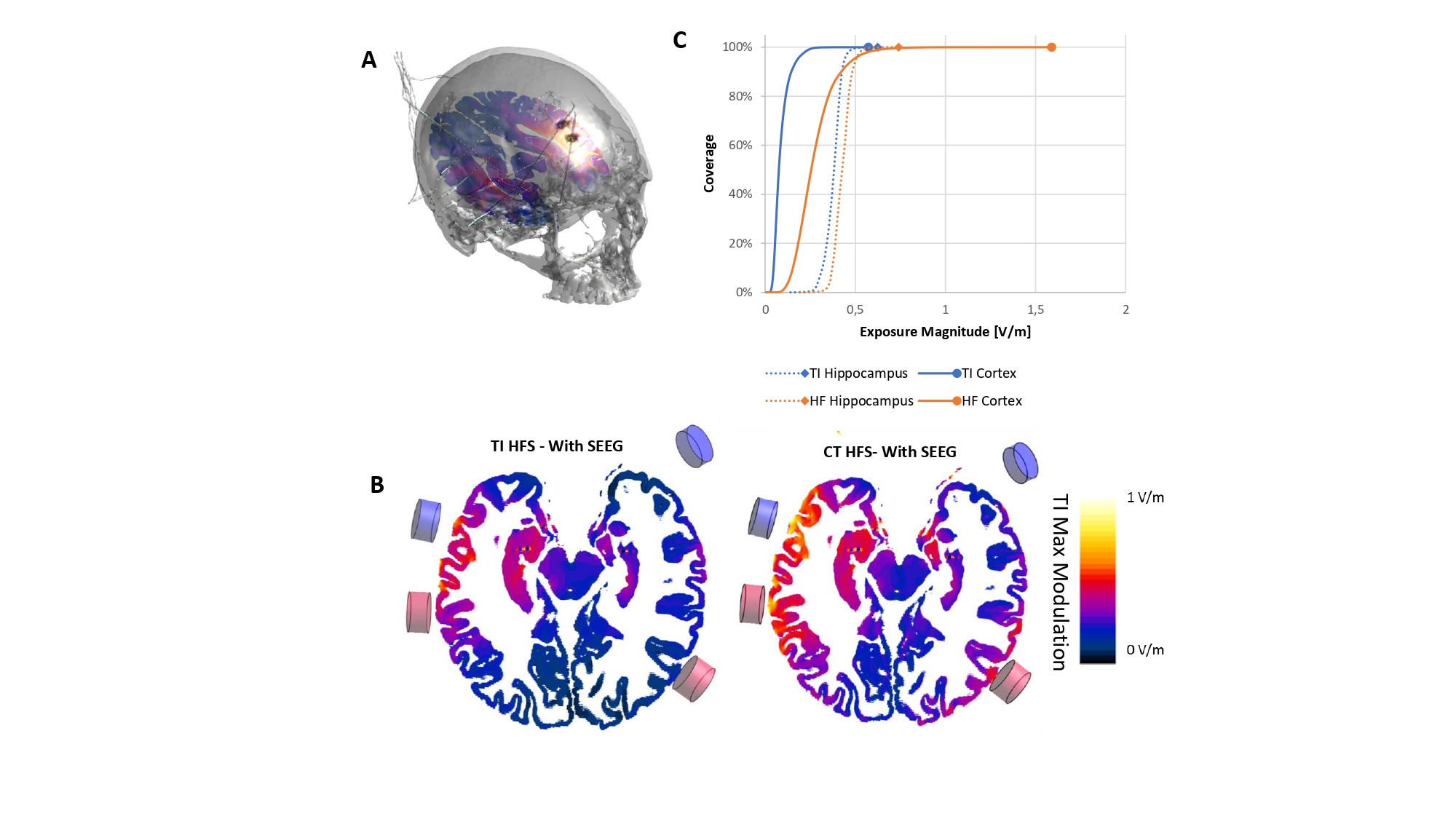

Supplement: Supplementary Figure 2 — EM simulations reveal highly localized field enhancement by the presence of metallic SEEG electrodes. (A) Illustration of the head models with the SEEG leads and the emplacement of the stimulation electrodes. (B) Simulated temporal interference envelope modulation amplitude distributions (left: TI along the direction of maximal modulation) and peak carrier field magnitude (right) in the presence of the SEEG leads, at a normalized current of 1 mA per channel. (C) Cumulative histograms of the cortical and hippocampal TI modulation and peak carrier distributions. [file Image_2.JPEG]
